# Influence of Liver Triglycerides on Suppression of Glucose Production by Insulin in Men

**DOI:** 10.1210/jc.2014-2404

**Published:** 2014-09-24

**Authors:** Eunsook S. Jin, Magdalene Szuszkiewicz-Garcia, Jeffrey D. Browning, Jeannie D. Baxter, Nicola Abate, Craig R. Malloy

**Affiliations:** Advanced Imaging Research Center (E.S.J., J.D.Br., J.D.Ba., C.R.M.), University of Texas Southwestern Medical Center, Dallas, Texas 75390-8568; Department of Internal Medicine (E.S.J., M.S.-G., J.D.B., C.R.M.), University of Texas Southwestern Medical Center, Dallas, Texas 75390; Department of Medicine (N.A.), Division of Endocrinology, University of Texas Medical Branch at Galveston, Texas 77555; Department of Radiology (C.R.M.), University of Texas Southwestern Medical Center, Dallas, Texas 75390; and VA North Texas Health Care System (C.R.M.), Dallas, Texas 75216

## Abstract

**Context:**

The ability of insulin to suppress hepatic glucose production is impaired among subjects with increased intrahepatic triglycerides (IHTG). However, little is known about the roles of insulin on the supporting fluxes of glucose production among patients with fatty liver.

**Objective:**

To evaluate the effects of insulin on fluxes through the three potential sources of plasma glucose (glycerol, the citric acid cycle, and glycogen) among patients with fatty liver.

**Design, Settings, Participants, and Intervention:**

Nineteen men with a range of IHTG (∼0.5% to 23%) were studied after an overnight fast and during hyperinsulinemia using magnetic resonance spectroscopy and stable isotope tracers.

**Main Outcome Measures:**

IHTG, gluconeogenesis from glycerol, gluconeogenesis from the citric acid cycle, glycogenolysis, and ^13^C-labeled glucose produced from the citric acid cycle during hyperinsulinemia were measured.

**Results:**

Men with high IHTG had higher fluxes through all pathways contributing to glucose production during hyperinsulinemia, compared to men with low IHTG, but they had similar fluxes after the fast. Consequently, men with fatty liver had impaired insulin efficiency in suppressing total glucose production as well as fluxes through all three biochemical pathways contributing to glucose. The detection of glucose isotopomers with ^13^C arising from [U-^13^C_3_]propionate ingested during hyperinsulinemia demonstrated continuous gluconeogenesis from the citric acid cycle in all subjects.

**Conclusions:**

These findings challenge the concept that individual glucose production pathways are selectively dysregulated during hepatic insulin resistance. Overproduction of glucose during hyperinsulinemia in men with fatty liver results from inadequate suppression of all the supporting fluxes of glucose production in response to insulin.

Hepatic insulin resistance is defined as excess glucose production by the liver during hyperinsulinemia (1, 2). The rate of glucose production is the sum of fluxes through gluconeogenesis from citric acid cycle intermediates and glycerol as well as glycogenolysis. Because flux through each of these three pathways is sensitive to multiple levels of regulation including expression of putative rate-controlling enzymes specific for each pathway, it is plausible that flux through one pathway or another may be selectively increased among patients likely to have hepatic insulin resistance (ie, fatty liver or type 2 diabetes). Intrahepatic triglycerides (IHTG) may be an important variable in hepatic insulin resistance (1–4), and excess triglycerides may be associated with generation of lipid-derived signaling molecules that inhibit insulin action (5). However, little is known about the pathways of hepatic glucose production among patients with excess IHTG. Under postabsorptive conditions, there was little effect of IHTG on either glycogenolysis or gluconeogenesis (6, 7), but the influence of IHTG on glucose production pathways during hyperinsulinemia has not been examined. In this study, we investigated fluxes from all three sources of glucose production in men with fatty liver under both fasting and hyperinsulinemic conditions.

## Subjects and Methods

### Research design

This study was approved by the Institutional Review Board at the University of Texas Southwestern Medical Center. Each participant provided written informed consent before participation. To achieve a range of IHTG, both lean and obese men (body mass index [BMI], 20–40 kg/m^2^; ages, 30–60 y) were recruited. Nineteen men finished all the procedures; they were Caucasians (n = 12), African Americans (n = 5), and Asians (n = 2). Women were excluded to avoid potential complexity in gender differences in insulin sensitivity (8, 9). Subjects with high blood pressure (>130/>85), any chronic illness, or a previous diagnosis of diabetes were excluded. No subjects were taking hypoglycemic agents or insulin. At the screening visit, fasting plasma glucose was measured in all subjects. Fasting glucose was < 125 mg/dL in all subjects, excepting one subject with a fasting glucose of 154 mg/dL. A 75-g oral glucose tolerance test was performed in all subjects, except the subject with fasting hyperglycemia. All participants ate a standardized diet (2100 cal/d; 30% protein, 30% fat, and 40% carbohydrates) for 3 days before the study.

**Figure 1.**
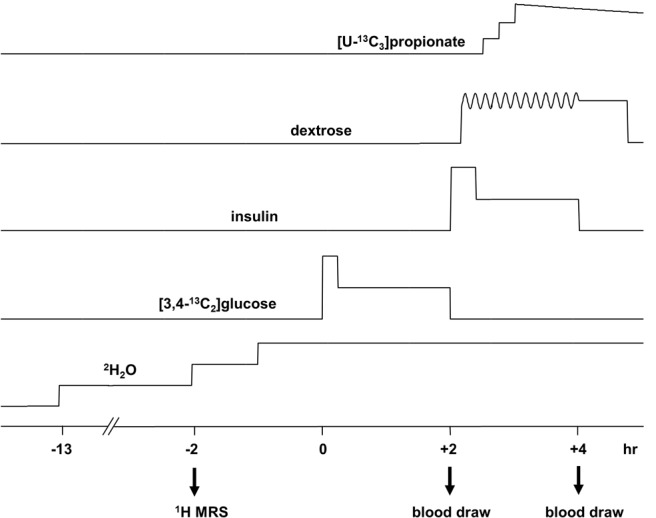
Schematic showing overall procedures. Before [3,4-^13^C_2_]glucose infusion, ^1^H MRS of liver was acquired, and three aliquots of ^2^H_2_O were ingested. Primed [3,4-^13^C_2_]glucose was infused at a constant rate for 2 hours, followed by the H-E clamp for 2 hours with primed insulin infusion at a rate of 80 mU/m^2^/min. Dextrose was infused during the clamp, and the infusion rate was adjusted to maintain euglycemia. Three aliquots of [U-^13^C_3_]propionate were ingested at t = 10, 30, and 50 minutes of the H-E clamp. Blood was drawn at the end of [3,4-^13^C_2_]glucose infusion under fasting and at the end of the H-E clamp for NMR analysis of glucose.

The overall study design is illustrated in Figure 1. Participants ate a meal at 6:30 pm before an overnight fast and received the first dose of ^2^H_2_O (70%, equally divided in three doses) orally at 9 pm. At 7:30 am the next day, subjects were admitted to the Advanced Imaging Research Center located on the North Campus of UT Southwestern. After receiving the second dose of ^2^H_2_O, subjects were directed to a magnetic resonance (MR) scanner room at 8 am for ^1^H-MR spectroscopy (^1^H-MRS) to measure IHTG. After the scan, they received the third dose of ^2^H_2_O at 9 am. The total dose of ^2^H_2_O was 5 g/kg body water (calculated as 60% of body weight), with a target of 0.5% enrichment in body water. Two iv catheters were placed. One catheter was positioned in an antecubital vein to infuse [3,4-^13^C_2_]glucose under the fasted, basal condition, and 20% dextrose and insulin under the hyperinsulinemic-euglycemic (H-E) clamp. A dorsal hand retrograde vein catheter was placed to draw blood. The hand with a catheter was kept in a hot box at 70°C for arterialization of venous blood. At 09:40 and 09:50 am, blood (1 mL) was drawn for baseline glucose measurement. At 10 am, blood (10 mL) was drawn again for insulin infusate preparation, glucose measurement, and blood chemistry analysis. Immediately after the blood draw, subjects received a bolus infusion of [3,4-^13^C_2_]glucose (6.209 μmol/kg bolus), followed by continuous infusion of the tracer (0.028 μmol/kg/min) for 2 hours. At the end of the 2-hour infusion, blood (30 mL) was drawn for nuclear MR (NMR) analysis of glucose. At 12:30 pm, the H-E clamp was initiated. A primed continuous infusion of regular human insulin was started at a rate of 80 mU/m^2^ body surface area/min for 2 hours. A 20% dextrose infusion was started at t = +4 minutes of the clamp, and the infusion rate was adjusted to maintain euglycemia. Every 5 minutes during the clamp, 1-mL blood was drawn to monitor glucose concentration. At t = +10, +30, and +50 minutes of the clamp, subjects ingested [U-^13^C_3_]propionate capsules (400 mg × 3 = 1200 mg). At t = +100 and +110 minutes, 10-mL blood was drawn for blood chemistry analysis. At t = +120 minutes, 40-mL blood was drawn for NMR analysis of glucose. Urine specimens were collected for determination of urinary glucose loss during the clamp.

### ^1^H-MRS for IHTG measurement and ^13^C-NMR for plasma glucose analysis

IHTG were measured by ^1^H-MRS using a 3.0 Tesla Achieva whole-body MR system (Philips Medical Systems). For single-voxel-localized data acquisition, a 2 × 2 × 2-cm^3^ voxel was positioned in the right hepatic lobe, avoiding major blood vessels, intrahepatic bile ducts, and the lateral margins of liver. The stimulated-echo acquisition mode (STEAM) sequence was applied without suppression of water signal. ^1^H-MR spectra were acquired with number of scans = 16, interpulse delay = 1.6 seconds, spin echo time = 14 milliseconds, mixing time = 18 milliseconds, and 2048 data points over a 1500-Hz spectral width. The levels of IHTG were calculated from the area of methylene resonance with respect to that of water resonance as described previously (7). Based on triglyceride contents in liver, men were divided into the low IHTG (≤ 5.5%; n = 13) or high IHTG (>5.5%; n = 6) group. Men with more than 5.5% IHTG were considered to have fatty liver (10). Blood glucose was converted to monoacetone glucose (MAG) for ^13^C-NMR acquisition as described previously (11).

### Glucose production from glycogen, glycerol, and the citric acid cycle

Endogenous glucose production (EGP) after an overnight fast was estimated from the dilution of infused [3,4-^13^C_2_]glucose using ^13^C-NMR analysis of MAG derived from glucose (12). EGP during the H-E clamp was estimated from the dilution of ^2^H enrichment in glucose H2 position as follows. ^2^H enrichment in glucose H2 incorporated only under the clamp (^2^H-glucose [during clamp]) was calculated using the following equation: ^2^H-glucose (during clamp) = ^2^H-glucose (clamp) − *F* × ^2^H-glucose (basal), where ^2^H-glucose (clamp) is ^2^H excess enrichment in glucose H2 at the end of the clamp, and ^2^H-glucose (basal) is the enrichment at the end of basal infusion. *F* is the fraction of circulated glucose from the basal condition to the clamp, and it was estimated by residual [3,4-^13^C_2_]glucose under the clamp: *F* = [3,4-^13^C_2_]glucose (clamp)/[3,4-^13^C_2_]glucose (basal), where [3,4-^13^C_2_]glucose (clamp) is excess [3,4-^13^C_2_]glucose enrichment at the end of the clamp and [3,4-^13^C_2_]glucose (basal) is the enrichment at the end of basal infusion. EGP under the clamp was then obtained using the following equation:

H2-glucose (during clamp)H2−glucose (basal)=EGPGinf+EGP.

where G_inf_ is an average glucose infusion rate during 80–120 minutes of the clamp.

**Figure 2.**
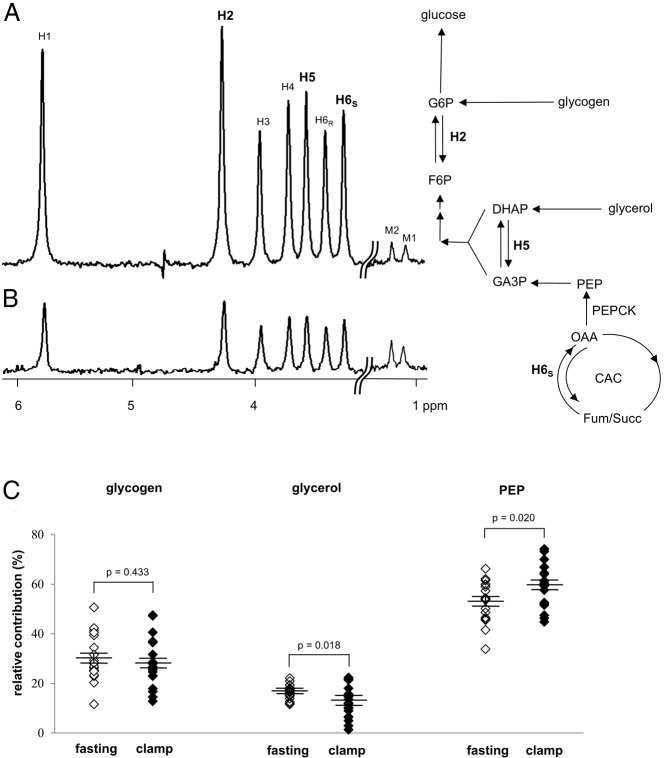
^2^H NMR spectra of MAG derived from blood glucose and relative contributions of glycogen, glycerol, and PEP to glucose production. Both spectra are from a single subject after an overnight fast (A) and under the H-E clamp (B). The hydrogen sites in glucose are labeled from 1–6 (H1, H2, etc) in the spectra. In the presence of ^2^H_2_O, glucose released from the liver may be enriched at positions H2 (G6P ↔ F6P), H5 (DHAP ↔ GA3P) and H6_S_ (OAA ↔ Fum), depending on the sources. The relative ^2^H enrichments in positions H2, H5, and H6_S_ indicate the fractional contributions of glycogen, glycerol, and the citric acid cycle (or PEP) to glucose production. The signal in the H6_S_ position indicates the presence of glucose derived from the citric acid cycle. The difference in ^2^H enrichments in positions H5 and H6_S_ indicates the contribution of glycerol, and the difference in positions H2 and H5 indicates the contribution from glycogen. There were no differences in relative contributions from three sources between low and high IHTG groups. The relative contribution from glycogen after an overnight fast remained the same under the H-E clamp. The relative contribution from glycerol under the clamp was decreased compared to that after an overnight fast, whereas the relative contribution from the citric acid cycle was increased (C). M1 and M2 in the spectra are the methyl resonances of MAG, which are natural abundance. Because the methyl groups were added during glucose conversion to MAG, their enrichments are independent of metabolism and can be used as references to estimate ^2^H enrichments of glucose. G6P, glucose 6-phosphate; F6P, fructose 6-phosphate; OAA, oxaloacetate; Succ, succinate; CAC, citric acid cycle; Fum, fumarate; DHAP, dihydroxyacetone phosphate; GA3P, glyceraldehyde 3-phosphate.

The relative contributions from glycogen, glycerol, and the citric acid cycle into glucose production were estimated using ^2^H enrichments at positions 2, 5, and 6_S_ of glucose (13–15), as determined from ^2^H-NMR spectra (Figure 2): flux from glycogen = EGP · (H2 − H5)/H2; flux from glycerol = 2 · EGP · (H5 − H6_S_)/H2; and flux from the citric acid cycle = 2 · EGP · H6_S_/H2.

### Metabolite and hormone assays

Glucose was assayed using glucose oxidase method (YSI 2300 Glucose Analyzer; GMI, Inc). Glycated hemoglobin (HbA1c), triglycerides, cholesterol, and other routine laboratory tests were performed by a commercial laboratory (Quest Diagnostics). Plasma insulin and C-peptide were determined by ELISA assay kits (Millipore Co. and ALPCO, respectively). Plasma nonesterified fatty acids (NEFAs) were assayed using the Vitros 250 analyzers (Johnson & Johnson), and free glycerol was determined using a commercial kit (Sigma).

### Insulin resistance index

An index of hepatic insulin resistance (16) was calculated by multiplying EGP and fasting plasma insulin (FPI). Homeostasis model assessment-estimated insulin resistance (HOMA-IR) was calculated by multiplying FPI and fasting plasma glucose, divided by the constant 22.5 (17).

### Statistical analysis

The data are expressed as mean ± SE. Comparisons between groups were performed using one-way ANOVA. The difference in mean values was considered statistically significant at a probability level of less than 5% (*P* < .05).

## Results

### Association of fatty liver with clinical variables and insulin resistance

The average IHTG were 1.3 ± 0.3% for the low IHTG group and 13.1 ± 2.4% for the high IHTG group. Age and levels of HbA1c, total cholesterol, high-density lipoprotein-cholesterol, low-density lipoprotein-cholesterol, glycerol, aspartate aminotransferase, and alanine aminotransferase were not significantly different between the groups. However, men with high IHTG had higher BMI, FPI, C-peptide, glucose, the molar ratio of C-peptide/insulin, NEFAs, and triglyceride levels compared with men with low IHTG (Table 1). After 2 hours of an oral glucose (75 g) load, plasma glucose levels were higher in men with high IHTG showing impaired glucose tolerance. In addition, men with high IHTG had higher HOMA-IR and an index of hepatic insulin resistance (16) calculated by EGP × FPI (Table 1).

**Table 1.** Clinical and Biochemical Characteristics of Subjects After an Overnight Fast and Under the H-E Clamp

Characteristics	Low IHTG (≤5.5%)	High IHTG (>5.5%)	*P* Value
IHTG, %	1.3 ± 0.3	13.1 ± 2.4	<.001
Age, y	45 ± 2	44 ± 4	.932
BMI, kg/m^2^	26.1 ± 0.9	32.6 ± 1.4	.001
2-h OGTT (glucose), mmol/L	6.5 ± 0.4	8.5 ± 0.8	.022
Hepatic IR (EGP × FPI)	1.0 ± 0.1	3.2 ± 0.6	<.001
HOMA-IR	0.7 ± 0.1	2.8 ± 0.7	<.001
Plasma under fasting			
Glucose, mmol/L	4.7 ± 0.1	5.8 ± 0.6	.015
HbA1c, %	5.4 ± 0.1	5.9 ± 0.5	.124
Triglycerides, mmol/L	1.1 ± 0.2	1.8 ± 0.3	.043
Total cholesterol, mmol/L	4.4 ± 0.3	4.8 ± 0.4	.437
HDL-cholesterol, mmol/L	1.2 ± 0.1	1.1 ± 0.1	.340
LDL-cholesterol, mmol/L	2.7 ± 0.2	2.9 ± 0.4	.676
AST, U/L	23 ± 3	28 ± 7	.511
ALT, U/L	22 ± 4	41 ± 14	.107
Insulin, μU/mL	3.2 ± 0.4	10.7 ± 1.8	<.001
C-peptide, ng/mL	0.42 ± 0.08	2.44 ± 0.96	.006
C-peptide:insulin molar ratio	4.6 ± 0.7	9.0 ± 2.5	.035
Glycerol, mmol/L	0.16 ± 0.01	0.18 ± 0.02	.439
NEFAs, mmol/L	0.99 ± 0.04	1.17 ± 0.05	.020
Plasma under the H-E clamp			
Glucose, mmol/L	4.7 ± 0.1	5.1 ± 0.1	.058
Insulin, μU/mL	104.2 ± 4.9	105.2 ± 12.0	.927
C-peptide, ng/mL	0.16 ± 0.03	0.98 ± 0.57	.044
Glycerol, mmol/L	0.12 ± 0.01	0.14 ± 0.02	.433
NEFAs, mmol/L	0.52 ± 0.01	0.59 ± 0.03	.015

Abbreviations: OGTT, oral glucose tolerance test; HDL, high-density lipoprotein; LDL, low-density lipoprotein; AST, aspartate aminotransferase; ALT, alanine aminotransferase; IR, insulin resistance. Values are mean ± SE for men with low IHTG (n = 13) and men with high IHTG (n = 6).

Insulin infusion during the clamp increased plasma insulin more than 10-fold over basal levels, and the concentrations achieved during the clamp were not different in men regardless of IHTG content: 104.2 ± 4.9 μU/mL in the low IHTG group, and 105.2 ± 12.0 μU/mL in the high IHTG group. Plasma glucose levels remained constant during the clamp in all subjects, and the levels were not statistically different between the groups (Supplemental Figure 1A). The G_inf_ necessary to maintain euglycemia during 80–120 minutes of the clamp was lower in men with high IHTG (Supplemental Figure 1, B and C). All the participants had only tracer amounts of urinary glucose (≤ 0.6 mmol/L) during the clamp.

### Glucose production pathways during a fast in men with fatty liver

The fractional contributions of glycogen, glycerol, and the citric acid cycle were determined from the relative ^2^H enrichments in H2, H5 and H6_S_ of glucose. Because any glucose carbons originating from the citric acid cycle must pass through phospho*enol*pyruvate (PEP), flux from PEP is equivalent to flux from the citric acid cycle. A typical ^2^H-NMR spectrum of MAG derived from glucose after an overnight fast is shown in Figure 2A. The large signal at the H6_S_ position indicates that a substantial fraction of glucose was derived from the citric acid cycle. The difference between ^2^H enrichments in positions H2 and H5 demonstrates that a significant fraction was derived from glycogen. By comparison, the ^2^H enrichment difference between H5 and H6_S_ indicates that the glycerol contribution was modest compared to glycogenolysis or gluconeogenesis from PEP. There were no differences in relative contributions from three sources between low and high IHTG groups. After an overnight fast, the fraction of glucose derived from PEP (53 ± 2%), glycogen (30 ± 2%), and glycerol (17 ± 1%) was similar across all subjects, regardless of IHTG levels.

**Figure 3.**
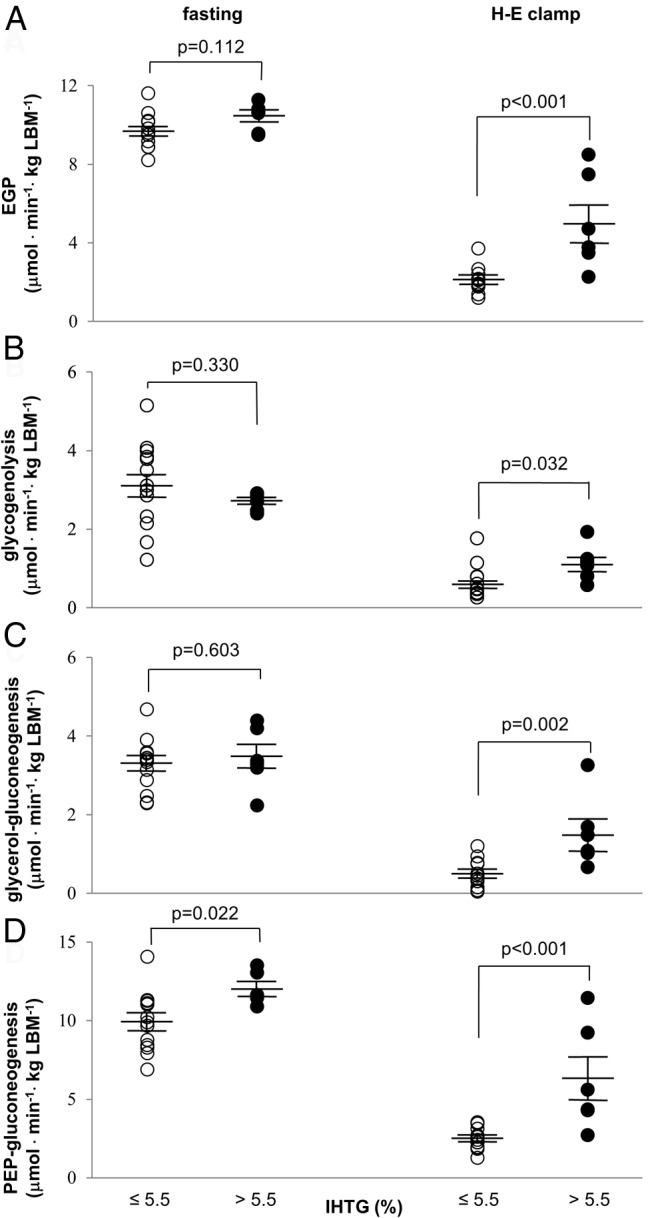
Fluxes through glucose production pathways in men with fatty liver. After an overnight fast, basal EGP and all supporting fluxes are similar in all men, except for the slight increase in gluconeogenesis from the citric acid cycle in men with fatty liver. Under the H-E clamp, men with fatty liver have significantly higher EGP (A) and all supporting fluxes: glycogenolysis (B), gluconeogenesis from glycerol (C), and gluconeogenesis from the citric acid cycle intermediates (or PEP) (D). All the supporting fluxes of EGP during hyperinsulinemia are efficiently suppressed in men with low IHTG, but not in men with high IHTG.

The absolute rates of glucose production from PEP, glycogen, and glycerol were determined by multiplying individual fractional contributions and EGP (Figure 3). After an overnight fast, EGP, glycogenolysis, and gluconeogenesis from glycerol were essentially the same between low and high IHTG groups (Figure 3, A–C), but gluconeogenesis from PEP was slightly increased in men with high IHTG (Figure 3D).

### Glucose production pathways during hyperinsulinemia in men with fatty liver

^2^H enrichments in glucose during the H-E clamp were lower compared to those after the overnight fast due to exogenous glucose infusion and suppressed EGP. This is illustrated in Figure 2B, where the enrichments in all positions are clearly reduced compared to the natural abundance signals from two methyl groups of MAG. Small changes in relative contributions were detected during the clamp when compared with those under the fasted condition (Figure 2C); relative glycerol contribution was decreased (17 ± 1% → 13 ± 2%: *P* = .018), whereas relative PEP contribution was increased (53 ± 2% → 60 ± 2%; *P* = .020). Relative glycogen contribution remained the same (30 ± 2% → 27 ± 2%; *P* = .433). However, again the relative contributions of glycogen, glycerol, and PEP during the clamp were the same regardless of IHTG content.

In contrast with minimal differences under the fast, all fluxes of EGP and supporting pathways under the H-E clamp were higher in men with high IHTG compared to the low IHTG group (Figure 3). Insulin was not efficient in suppressing all the glucose production pathways in men with fatty liver.

### Persistent gluconeogenesis from the citric acid cycle during hyperinsulinemia

**Figure 4.**
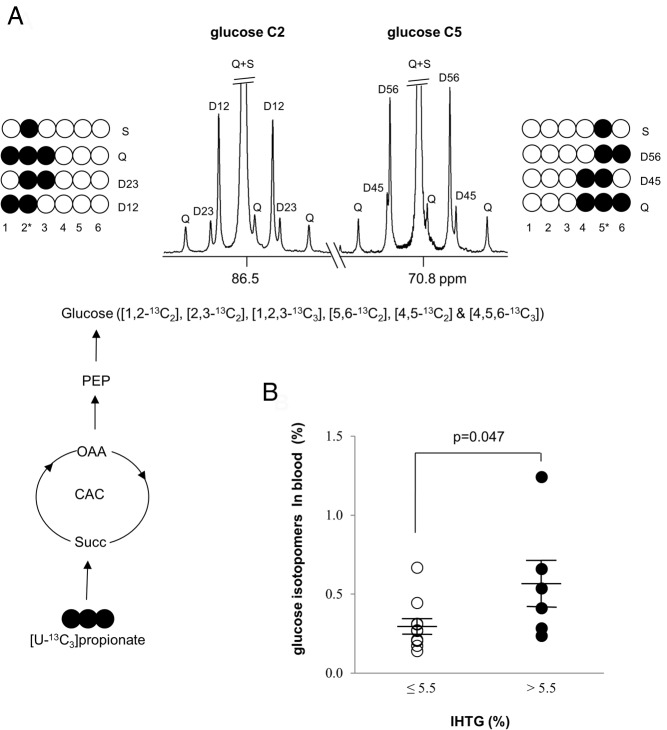
Glucose production from the citric acid cycle during hyperinsulinemia. The entry of [U-^13^C_3_]propionate into the citric acid cycle produces ^13^C isotopomers in all the intermediates of the cycle including oxaloacetate. The participation of ^13^C oxaloacetate isotopomers in gluconeogenesis produces ^13^C glucose isotopomers (A). A proton-decoupled ^13^C NMR spectrum of MAG derived from blood glucose (carbon 2 and 5 regions) during the H-E clamp shows ^13^C-^13^C spin-spin coupling that can be derived only from [U-^13^C_3_]propionate metabolized in the citric acid cycle. Men with high IHTG had higher enrichment by ^13^C glucose isotopomers (B). The enrichment is the sum of following glucose isotopomers in blood: [1,2-^13^C_2_]-, [2,3-^13^C_2_]-, [1,2,3-^13^C_3_]-, [4,5-^13^C_2_]-, [5,6-^13^C_2_]-, and [4,5,6-^13^C_3_]glucose. Q, quartet or doublet of doublets; D12, doublet due to J_12_; D23, doublet due to J_23_; D45, doublet due to J_45_; D56, doublet due to J_56_; S, singlet. ○, ^12^C; ●, ^13^C.

Propionate is metabolized primarily in the liver, where it enters the citric acid cycle through succinyl-coenzyme A (18, 19). The subsequent turnover in the cycle produces ^13^C isotopomers in all the intermediates of the cycle including oxaloacetate. The participation of ^13^C oxaloacetate isotopomers in gluconeogenesis through PEP carboxykinase (PEPCK) generates characteristic ^13^C labeling in blood glucose (Figure 4A). Note that the first dose of [U-^13^C_3_]propionate was ingested at 10 minutes of the clamp after the priming bolus infusion of insulin and was followed by the second dose and the final dose at 30 and 50 minutes, respectively, during the clamp (Figure 1).

The ^13^C labeling pattern in glucose (Figure 4A) is unequivocal evidence for gluconeogenesis from the citric acid cycle during hyperinsulinemia. All subjects showed glucose isotopomers with ^13^C originated from [U-^13^C_3_]propionate. The summed enrichment with [1,2-^13^C_2_]-, [2,3-^13^C_2_]-, [1,2,3-^13^C_3_]-, [4,5-^13^C_2_]-, [5,6-^13^C_2_]-, and [4,5,6-^13^C_3_]glucose in blood was approximately 0.2–1.2%. The summed enrichment in blood glucose was slightly higher in men with high IHTG (Figure 4B).

## Discussion

Analysis of glucose production in the liver in human subjects is challenging because of the capacity of a healthy liver to readily shift among multiple biochemical sources for exporting glucose after an overnight fast. In this study, ^13^C-labeled glucose was used for turnover calculations, and ^2^H_2_O was used simultaneously using a conceptual approach developed for mass spectrometry to assess the relative contributions of various pathways to EGP. In the current study, these ^13^C and ^2^H methods could be integrated because, unlike with mass spectrometry, it is a simple matter using NMR to measure position-specific ^13^C and ^2^H enrichment in glucose in the presence of both isotopes. This ability to monitor multiple reactions in glucose production simultaneously was coupled with conventional ^1^H-MRS of liver fats to assess a possible interaction between IHTG and EGP pathways. There was minimal influence of IHTG on glucose production pathways after the overnight fast. However, men with fatty liver had substantially higher fluxes through all three pathways of EGP during hyperinsulinemia. Because EGP is primarily hepatic in origin, we conclude that hepatic insulin resistance in fatty liver is characterized by failure to suppress fluxes through all pathways of glucose production.

### Relative fluxes through glucose production pathways by ^2^H-NMR

For the measurement of relative glycogenolysis and gluconeogenesis in human subjects, Landau et al (13, 14) popularized the use of ^2^H_2_O followed by measurement of the ratio of ^2^H enrichment in H5 relative to H2 by mass spectrometry to assess combined gluconeogenesis from glycerol and the citric acid cycle. Although mass spectrometry offers high sensitivity, ^2^H-NMR allows measurement of ^2^H enrichments in all seven sites of glucose in a single experiment, and consequently, it is a simple matter to distinguish gluconeogenesis from glycerol vs gluconeogenesis from the citric acid cycle (15). Results from the current study are consistent with earlier reports of glucose production in postabsorptive conditions. Among healthy subjects, depending on the duration of fasting, glycerol contributes anywhere from approximately 5% to 22% of EGP (15, 20). We also found that relative glycerol contribution during hyperinsulinemia was less compared to a postabsorptive condition. In contrast, the relative PEP contribution was increased during hyperinsulinemia. This observation was interesting because the combined relative contribution from overall gluconeogenesis (ie, both glycerol and PEP) was not altered by hyperinsulinemia, although the relative contribution from glycerol alone was decreased whereas that from PEP was increased.

### Interaction between glucose production pathways and hepatic fats

Similar patterns of glucose production after an overnight fast were found regardless of IHTG content, as reported previously (6, 7). In the current study, only slightly increased gluconeogenesis from the citric acid cycle was observed in men with fatty liver. However, men with fatty liver were insulin resistant after an overnight fast because they had higher insulin levels, which was confirmed by an index of hepatic insulin resistance (ie, EGP × FPI) and HOMA-IR (Table 1).

In marked contrast to the minimal interaction between IHTG and EGP after an overnight fast, there were dramatic differences in EGP pathways during hyperinsulinemia in men depending on IHTG content. Because specific enzyme-catalyzed reactions are thought to play a major role in the regulation of EGP through various pathways in response to insulin (21), a popular concept is that hepatic glucose overproduction in insulin-resistant states reflects excess flux in a specific pathway. The design of this experiment—simultaneous measurement of fluxes in all pathways—enabled evaluation of this concept. When analyzed using IHTG as an independent variable, hepatic insulin resistance in fatty liver was due to blunted sensitivity to insulin in suppressing fluxes through all pathways of glucose production rather than persistent flux through a specific pathway.

However, this observation does not mean that fluxes through all pathways are quantitatively of equal importance in excess EGP during hyperinsulinemia. Specifically, PEPCK is thought to play a key role in regulation of EGP because it converts oxaloacetate to PEP, the first step of gluconeogenesis from the citric acid cycle. Quantitatively, the relative contribution of gluconeogenesis from the citric acid cycle was higher compared to either gluconeogenesis from glycerol or glycogenolysis. Thus, gluconeogenesis from the citric acid cycle played a more significant role after an overnight fast compared to the other fluxes. This observation after a 15-hour fast may not be applicable in postprandial conditions or after a shorter fast, where we expect increased contribution from glycogen (and decreased contribution from gluconeogenesis) due to abundant glycogen in the liver.

### Gluconeogenesis from the citric acid cycle during hyperinsulinemia

Although a lower rate of insulin infusion could be more appropriate to explore the effect of insulin on EGP, a higher rate (80 mU/m^2^/min) was chosen in the current study to evaluate whether gluconeogenesis through the citric acid cycle persists even at high plasma insulin levels. The detection of glucose isotopomers with ^13^C from [U-^13^C_3_]propionate is direct evidence for continuous gluconeogenesis from the citric acid cycle during hyperinsulinemia (plasma insulin > ∼100 μU/mL). The glucose isotopomers monitored in the current study (ie, [1,2-^13^C_2_]-, [2,3-^13^C_2_]-, [1,2,3-^13^C_3_]-, [4,5-^13^C_2_]-, [5,6-^13^C_2_]-, and [4,5,6-^13^C_3_]glucose) must originate exclusively from [U-^13^C_3_]propionate. These multiply-enriched isotopomers of glucose cannot be generated from [3,4-^13^C_2_]glucose infusion during the basal period. Glycolysis of [3,4-^13^C_2_]glucose produces trioses with single-labeled isotopomers on either the carbon 1 or 3 position. When the single-labeled trioses participate in gluconeogenesis, it will regenerate single-labeled glucose isotopomers on the carbon 3 or 4 position. Single-labeled glucose isotopomers were excluded in the estimation of plasma glucose from [U-^13^C_3_]propionate because glucose labeled in a single carbon may arise from natural abundance ^13^C or from metabolism of [3,4-^13^C_2_]glucose.

Glucose isotopomers with ^13^C from [U-^13^C_3_]propionate were observed in all men. Glucose molecules arising from propionate were present in higher enrichment in blood glucose in men with fatty liver. This observation could be explained by two factors: the lower rate of glucose infusion during the H-E clamp, and higher gluconeogenesis from the citric acid cycle in men with high IHTG measured by ^2^H enrichments of glucose. Gluconeogenesis during hyperinsulinemia is generally thought to be completely suppressed. Basu et al (22) observed continuous gluconeogenesis under the H-E clamp using the ^2^H_2_O method but cautioned that glycogen cycling and transaldolase reactions could cause overestimation of gluconeogenesis with the method. In the current study, the plasma insulin level was approximately 4-fold higher compared to the study of Basu et al (22). The observation of plasma glucose arising from [U-^13^C_3_]propionate demonstrates persistent gluconeogenesis from the citric acid cycle during hyperinsulinemia. Detection of multiply labeled plasma glucose under these conditions cannot arise as an artifact of either glycogen cycling or the transaldolase reaction, and consequently, the current results support the earlier conclusion (22). To our knowledge, this is the first study to provide a direct measure of glucose production in humans during the H-E clamp by administrating a tracer passing through the citric acid cycle.

We elected to stratify subjects according to IHTG. All subjects were studied with a 2-hour H-E clamp to determine the influence of IHTG on metabolism measured with this clamp protocol. It should be noted that slightly different results could occur with a 3-hour clamp. According to Doberne et al (23), a longer duration was reported to increase glucose utilization progressively up to 5 hours. Karelis et al (24) reported that insulin sensitivity was increased by approximately 10% with a 3-hour clamp compared to a 2-hour clamp. These observations suggest that EGP would be slightly lower in the current study if the clamp lasted longer. However, our conclusion of persistent EGP would almost certainly remain the same even with a longer H-E clamp.

### Interaction between IHTG and insulin secretion

After an overnight fast, plasma insulin and C-peptide levels were high in men with high IHTG. They also had a higher molar ratio of C-peptide/insulin, indicating higher hepatic insulin clearance in men with excess IHTG. The liver is the major organ responsible for insulin degradation, influencing insulin delivery to extrahepatic organs (25). Despite the higher hepatic clearance, insulin levels in periphery blood were higher in men with fatty liver, suggesting substantial insulin secretion in those men. Exogenous insulin supply during the H-E clamp decreased endogenous insulin production in all subjects, as evidenced by reduced C-peptide levels (Table 1), but men with high IHTG had higher C-peptide levels compared to men with low IHTG during the H-E clamp, again suggesting higher insulin secretion.

### Summary

Hepatic insulin resistance in men with high IHTG was characterized by impaired efficacy of insulin in suppressing all three sources of glucose production rather than reduced efficacy in suppressing a particular pathway. Our study supports the view that IHTG content is an important target of therapy, not only to prevent progression of liver disease but also to ameliorate systemic glucose metabolism.
